# Elevated CO_2_ accelerates polycyclic aromatic hydrocarbon accumulation in a paddy soil grown with rice

**DOI:** 10.1371/journal.pone.0196439

**Published:** 2018-04-24

**Authors:** Fuxun Ai, Nico Eisenhauer, Yuwei Xie, Jianguo Zhu, Alexandre Jousset, Wenchao Du, Ying Yin, Xiaowei Zhang, Rong Ji, Hongyan Guo

**Affiliations:** 1 Stake Key Laboratory of Pollution Control and Resource Reuse, School of Environment, Nanjing University, Nanjing, China; 2 German Centre for Integrative Biodiversity Research (iDiv) Halle-Jena-Leipzig, Leipzig, Germany; 3 Institute of Biology, Leipzig University, Leipzig, Germany; 4 State Key Laboratory of Soil and Sustainable Agriculture, Institute of Soil Science, Chinese Academy of Science, Nanjing, China; 5 Institute of Environmental Biology, Utrecht University, Utrecht, The Netherlands; Universite Paris-Sud, FRANCE

## Abstract

The concentration of atmospheric carbon dioxide (CO_2_) and polycyclic aromatic hydrocarbons (PAHs) contents in the environment have been rising due to human activities. Elevated CO_2_ (eCO_2_) levels have been shown to affect plant physiology and soil microbes, which may alter the degradation of organic pollutants. Here, we study the effect of eCO_2_ on PAH accumulation in a paddy soil grown with rice. We collected soil and plant samples after rice harvest from a free-air CO_2_ enrichment (FACE) system, which had already run for more than 15 years. Our results show that eCO_2_ increased PAH concentrations in the soil, and we link this effect to a shift in soil microbial community structure and function. Elevated CO_2_ changed the composition of soil microbial communities, especially by reducing the abundance of some microbial groups driving PAH degradation. Our study indicates that elevated CO_2_ levels may weaken the self-cleaning ability of soils related to organic pollutants. Such changes in the function of soil microbial communities may threaten the quality of crops, with unknown implications for food safety and human health in future climate scenarios.

## Introduction

Due to global industrialization and human population growth, atmospheric concentration of carbon dioxide (CO_2_) has raised from approximately 280 ppm in pre-industrial times to approximately 400 ppm today, and it is expected to continue increasing in the future [1]. Human activities have further caused a global contamination of soils with organic pollutants [2]. Among them, polycyclic aromatic hydrocarbons (PAHs) have prompted significant concern, due to their ubiquitous occurrence, recalcitrance, toxicity and bioaccumulation potential [3]. Although adsorption, volatilization and chemical degradation are involved during the removal process of PAHs from soils, biodegradation is the major degradation process of PAHs, which depends on soil microbial communities and environmental conditions [4].

Several anthropogenic changes in environmental conditions were shown to influence soil microbial communities and their biodegradation potential. For instance, high tropospheric O_3_ concentrations have been reported to decrease inputs and to change the composition of assimilates released into the rhizosphere [5], which in turn affects soil microbial communities. By contrast, higher plant diversity has been shown to increase rhizosphere carbon inputs into the soil microbial community resulting in an increased microbial diversity and activity [6]. Furthermore, both temperature and aridity regulate the spatial variability of soil multifunctionality [7]. Here, we focus on the effects of elevated CO_2_ concentrations on soil microbial communities and their role in biodegradation. Elevated CO_2_ concentrations (eCO_2_) are known to stimulate the photosynthesis of plants, enhance carbon inputs to the soil, and change the composition of root exudates released into the rhizosphere, thereby altering microbial composition and activity in soils [8]. As a consequence, we speculated that eCO_2_ changes the biomass and community composition of microbes and the environmental conditions in soil. These alterations were expected to affect the biodegradation process of PAHs in soil, and thus the accumulation potential of PAHs in soil and plants. We studied the effects of eCO_2_ on PAH degradation by assessing soil microbial community structure through high-throughput sequencing and mineralization of ^14^C-PAHs by fresh soils that had been conditioned by the different CO_2_ treatments. Results of this study will be helpful to understand and forecast the potential of PAHs accumulation in soils in future climate scenarios and how this may affect food safety and human health.

## Materials and methods

### FACE system

The FACE system was established in the town of Xiaoji, Jiangdu, Jiangsu Province, China (119°42’E, 32°35’N), in 2001, details about the FACE system were described previously [9]. In brief, the FACE system consists of six octagonal plots (diameter 14 m), three for ambient CO_2_ conditions (ambient plots, CO_2_ concentration at around 370 ppm reflecting the current local CO_2_ concentration), three for elevated CO_2_ conditions (FACE plots, CO_2_ concentration around 570 ppm reflecting predicted CO_2_ concentration in 2050 [1]). Ambient plots and FACE plots are arranged crosswise. For the study region, the annual mean temperature is 15°C, the annual precipitation is 980 mm, and the annual no-frost period is approximately 220 days. In the south of the FACE system, 200 m away there is a highway, in the west 1.5 km away there is an expressway, and in the east 2.0 km away there is a small town, and within 2 km around the FACE system there are several villages and factories. The sources of PAHs in this area are a mixture of pyrogenic and petrogenic compounds, with pyrogenic ones as main source [10]. Highways and factories are the main PAH sources to contaminate the soils in the investigated soil of farm fields [11].

### Sample collection

At the end of October in 2015 and 2016, three soil samples were collected randomly in each of the six plots (distance between sampling points more than 2 m), shortly after rice (*Oryza sativa* L. cv. Wu xiang jing 14) harvest. At each sampling point, three small columns (diameter 2 cm, distance between columns around 20 cm) were collected from the top 20 cm of the soil, mixed thoroughly, and separated into two halves; one half was stored at -20°C for PAH measurements, the other half was stored as fresh soils for soil microbial analysis. In 2016, plant samples were collected, by randomly sampling three ripe rice plants per plot (distance between sampling points more than 2 m). For each plant, grain husks were removed and seeds were stored at -20°C for PAH measurements.

### PAH determination

All samples for PAH measurements were freeze-dried, and then soil samples were homogenized and sieved through a 3-mm sieve; rice seeds were ground. Samples were extracted by applying an acceleration solvent comprised of 80% dichloromethane and 20% n-hexane. Extracts were purified and concentrated, and then analyzed on a gas chromatograph (Agilent Technologies 6890N Network GC System Agilent Co., USA) equipped with a mass selective detector mass spectrometer (Agilent 5973 Network).

### Analysis of soil microbial communities

After plant harvest, fresh soil samples for microbial community analysis were homogenized and sieved through a 3-mm sieve. Soil microbial community analysis was performed by high-throughput sequencing technique. Details about DNA extraction, PCR amplification, sequencing and sequencing data analysis were described previously [12].

### ^14^C-phenanthrene mineralization

Fresh soil samples for ^14^C-phenanthrene mineralization were also homogenized and sieved. Two g of each soil sample was weighed into a glass tube, soil moisture was adjusted to 40% by adding sterilized water, and 100 μL ^14^C-phenanthrene solution (radioactivity intensity was around 16,000 dpm) was sprayed onto the soil. The tube was sealed by a rubber plug adhered to a plastic vial (which could be directly placed in a liquid scintillation counter) containing 2 mL sodium hydroxide (1 M). The vial was replaced after a certain time, 1 mL cocktail (Gold Star, Meridian Biotechnologies Ltd, England) was added, capped and mixed thoroughly. Radioactivity of the solution was measured by a liquid scintillation counter (LS 6500, Beckman, USA).

### Statistical analysis

Given that three samples were taken from each plot (representing pseudo-replicates), we performed conservative analyses by calculating mean values per plot and performing one-way analysis of variance and covariance (ANOVA) with three replicates (= plots) per treatment. We tested treatment effects on the concentration of PAHs in rice seeds and soil (including different sources; pyrogenic and petrogenic), soil microbial community properties, and ^14^C-phenanthrene mineralization. Data were expressed as mean ± standard error (n = 3). Statistical differences between aCO_2_ and eCO_2_ treatments were significant when p < 0.05.

Sequence data were deposited into the NCBI Sequence Read Archive under accession number SRP136395.

## Results and discussion

**Fig 1:**
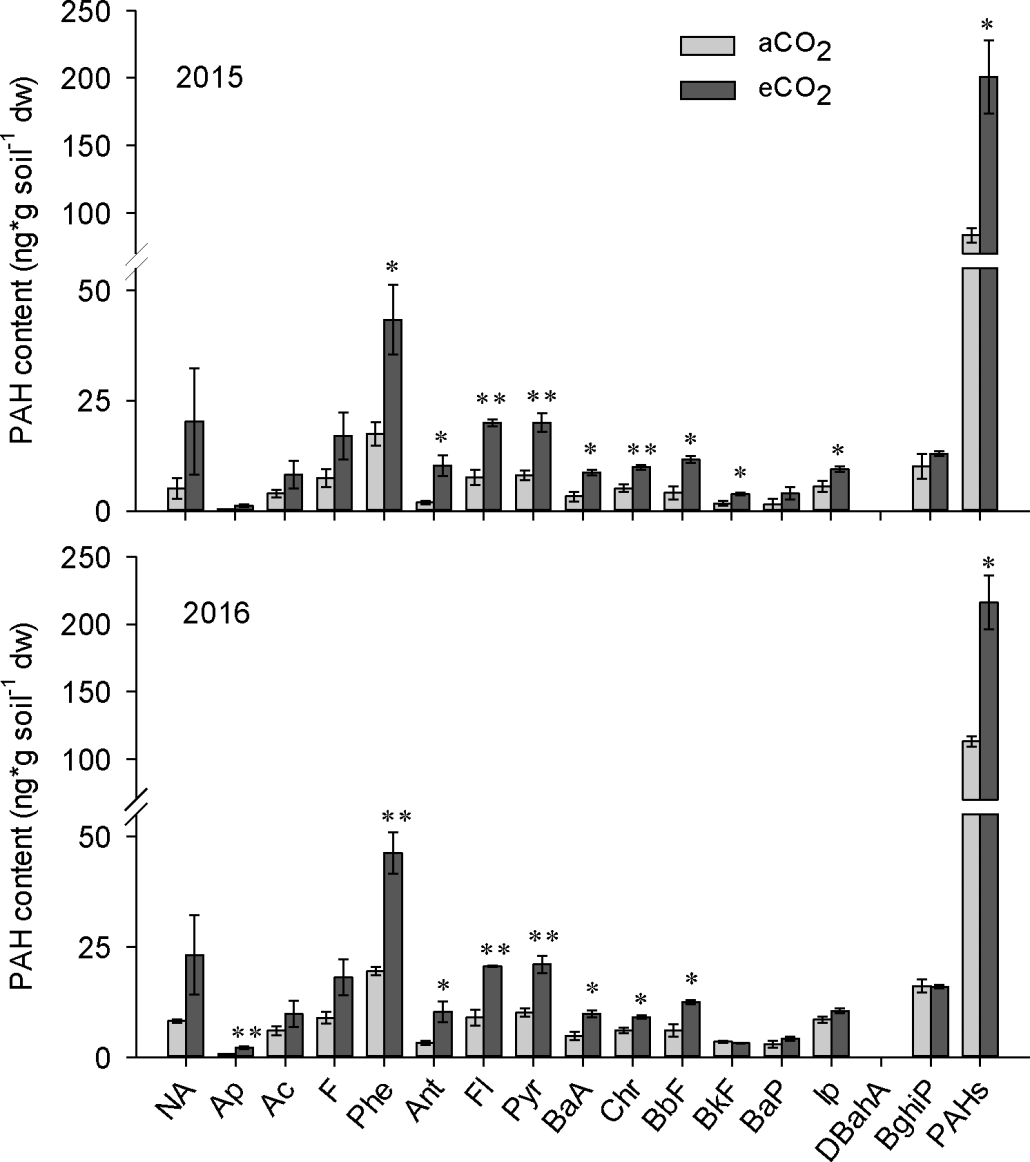
Contents of individual polycyclic aromatic hydrocarbons (PAHs) and total PAHs in soil at ambient (370 ppm) or elevated (570 ppm) CO_2_ levels in year 2015 and 2016. aCO_2_, ambient CO_2_; eCO_2_, elevated CO_2_. NA, AP, AC, F, Phe, Ant, Fl, Pyr, BaA, Chr, BbF, BkF, BaP, IP, DBahA, BghiP represent Naphthalene, Acenaphthylene, Acenaphthene, Fluorene, Phenanthrene, Anthracene, Fluoranthene, Pyrene, Benzo(a)anthracene, Chrysene, Benzo(b)fluoranthene, Benzo(k)fluoranthene, Benzo(a)pyrene, Indene(1,2,3-c,d)pyrene, Dibenzo(a,h)anthracene and Benzo(g,h,i)perylene, respectively. Data are means of three replicates ± standard error. Asterisks among columns indicate significant differences between aCO_2_ and eCO_2_ conditions (* indicate p < 0.05, ** indicate p < 0.01).

In both 2015 and 2016, the concentrations of the majority of the 16 PAHs listed as the US EPA priority pollutants [13] in soil were significantly higher at eCO_2_ than at aCO_2_ (Fig 1, S1 Table). The positive effect of eCO_2_ on the accumulation of PAHs from pyrogenic source was more significant than that from petrogenic source (S1 Table). This difference may due to the continuous inputs of PAHs from pyrogenic source, such as fossil fuel combustion in this area. Among all PAHs, anthracene was increased the most at eCO_2_ in 2015 (5.36-fold compared to aCO_2_), and acenaphthylene was increased the most at eCO_2_ in 2016 (3.37-fold compared to aCO_2_; S1 Table). Among all the 16 priority controlled PAHs in both aCO_2_ and eCO_2_ treatments, the concentration of phenanthrene in soil under eCO_2_ was highest, ranging between 43.30 μg kg^-1^ in 2015 and 46.27 μg kg^-1^ in 2016 (Fig 1). The total content of PAHs was increased 2.40-fold and 1.91-fold at eCO_2_ compared to aCO_2_ in 2015 and 2016, respectively (S1 Table).

**Fig 2:**
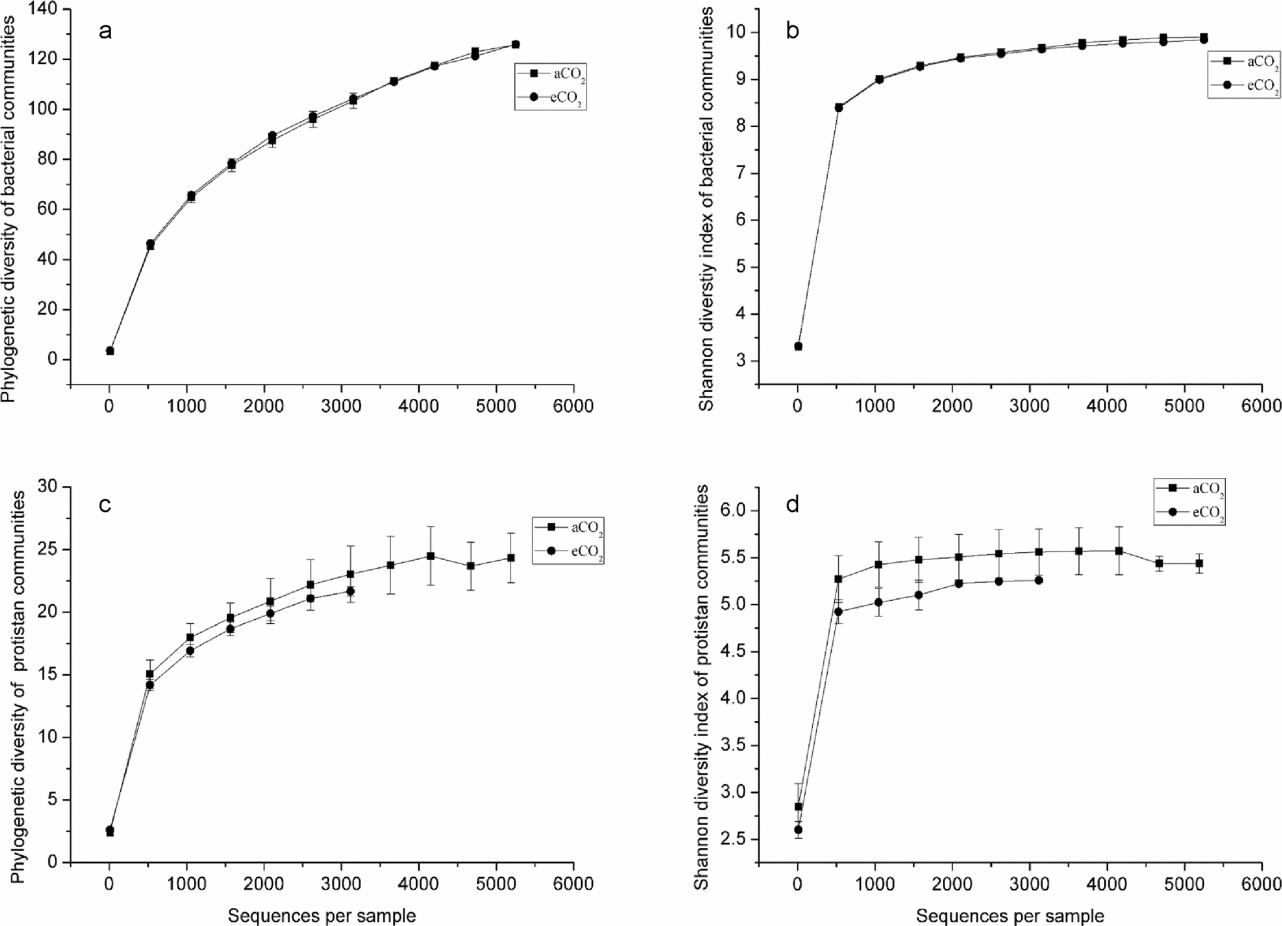
Phylogenetic diversity and richness (Shannon diversity index) of soil microbial communities as affected by atmospheric CO_2_ concentrations. a, phylogenetic diversity of bacterial communities; b, richness of bacterial phyla; c, phylogenetic diversity of protistan communities; d, richness of protistan classes. aCO_2_, ambient CO_2_ (370 ppm); eCO_2_, elevated CO_2_ (570 ppm).

**Fig 3:**
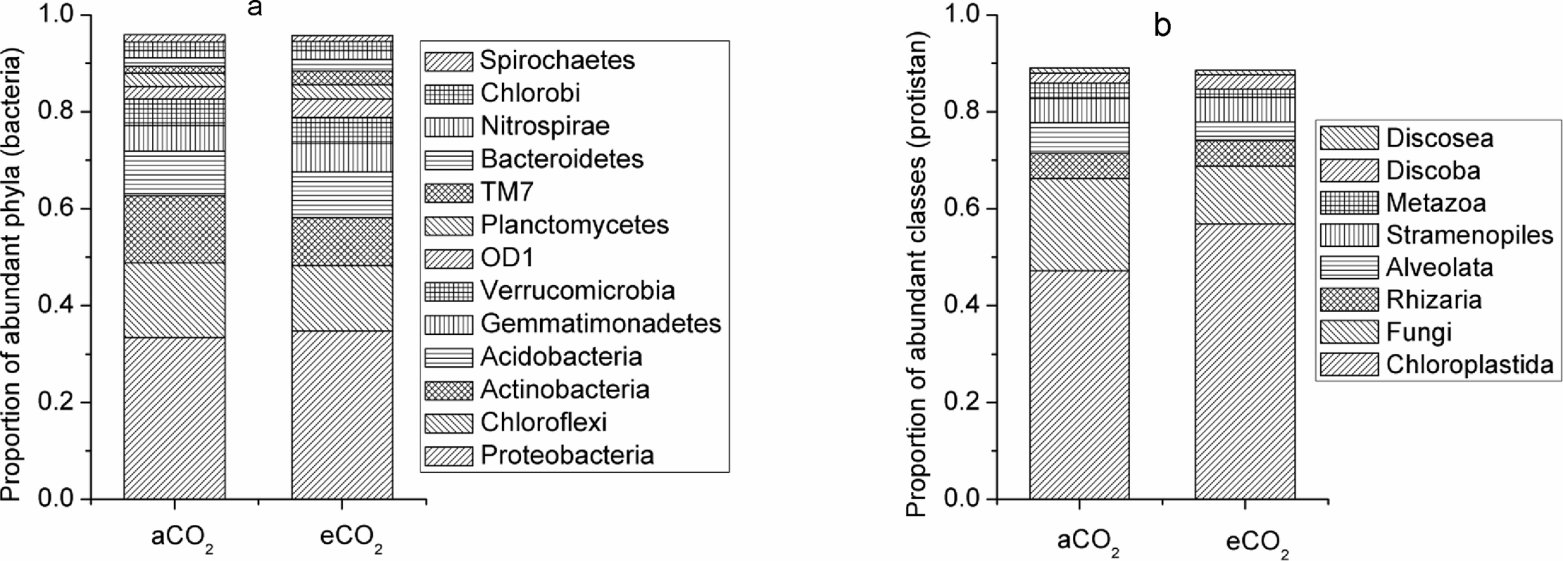
**Composition of bacterial communities at phyla level (a) and protistan communities at class level (b) of soils under ambient or elevated CO_2_ conditions**. aCO_2_, ambient CO_2_ (370 ppm); eCO_2_, elevated CO_2_ (570 ppm).

**Fig 4:**
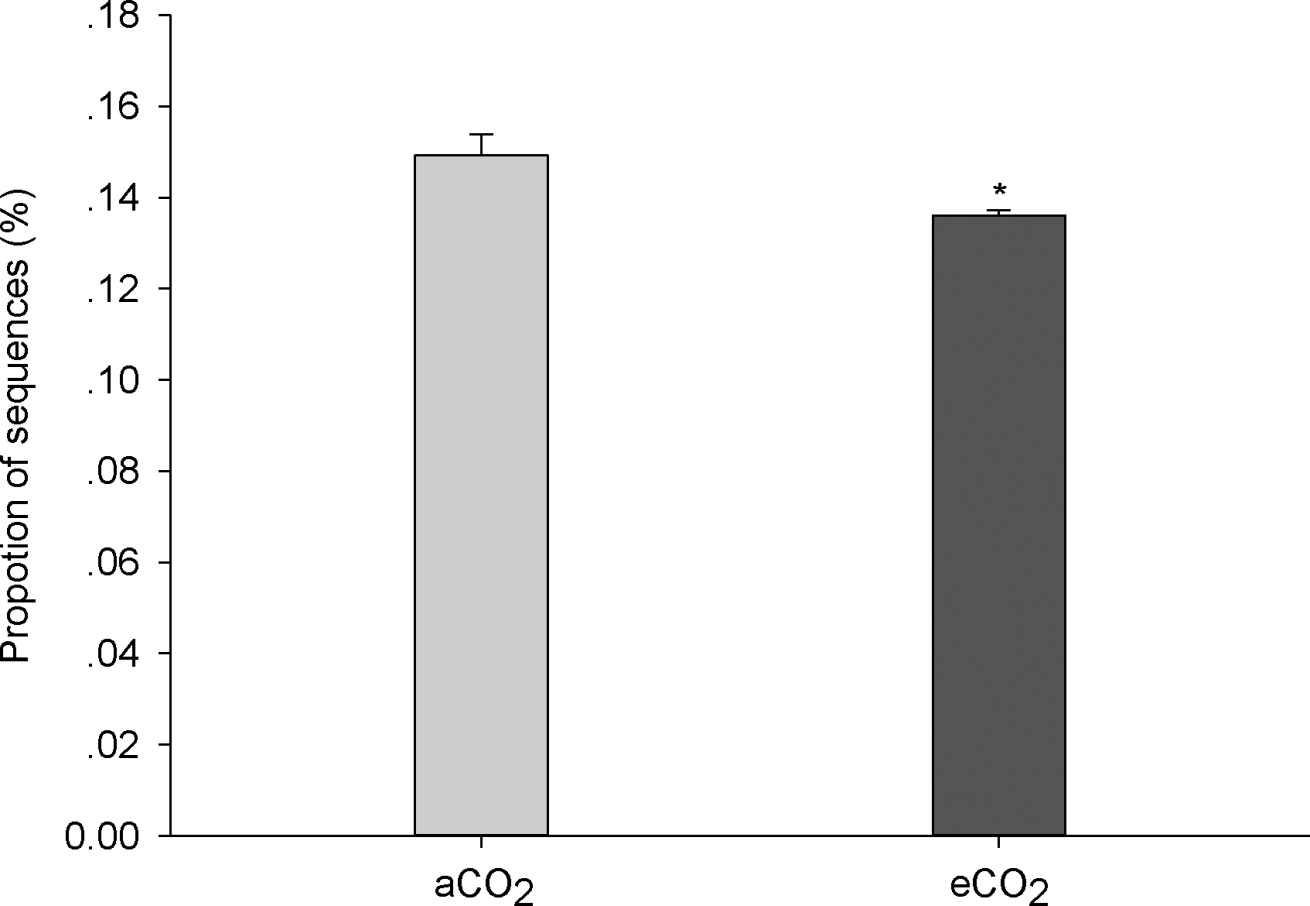
Proportion of bacterial sequences contributing to PAH degradation at ambient (370 ppm) or elevated (570 ppm) CO_2_ levels. aCO_2_, ambient CO_2_; eCO_2_, elevated CO_2_. Data are means of three replicates ± standard error. Asterisks among columns indicate significant differences between aCO_2_ and eCO_2_ conditions (p < 0.05).

**Fig 5:**
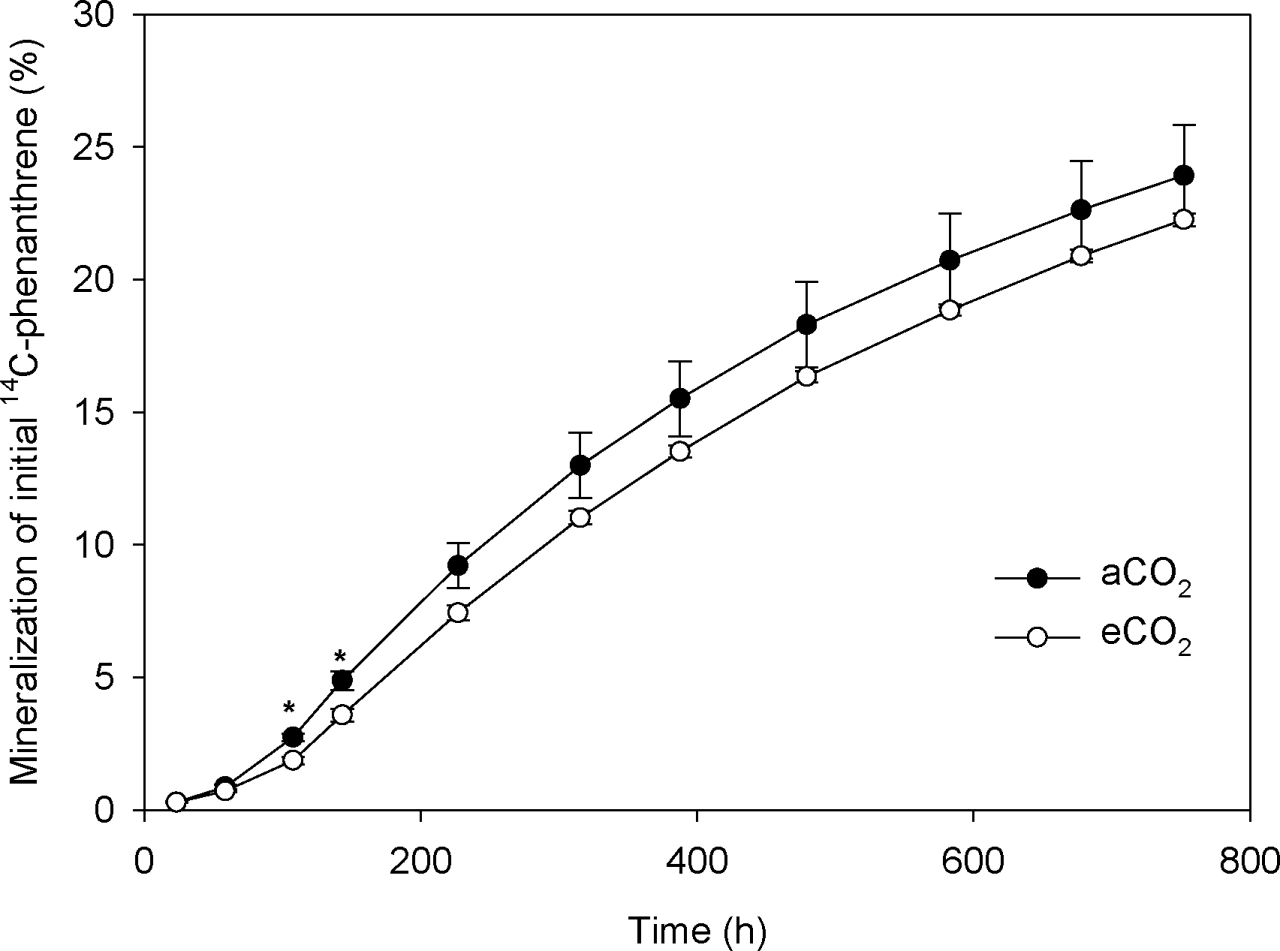
Mineralization of ^14^C-phenanthrene by fresh soils conditioned at ambient (370 ppm) or elevated (570 ppm) CO_2_ levels. aCO_2_, ambient CO_2_; eCO_2_, elevated CO_2_. Data are means of three replicates ± standard error. Asterisks among plots indicate significant differences between aCO_2_ and eCO_2_ conditions (p < 0.05).

Although few studies have focused on the effect of eCO_2_ on the environmental process of organic pollutant accumulation and degradation, there are some studies that investigated the effect of eCO_2_ on the environmental fate of heavy metals [9] and metallic oxide nanoparticles [14]. These studies showed that eCO_2_ changed the condition of soil and sediments (mainly by decreasing pH), increased the bioavailability of heavy metals, and thereby increased bioaccumulation of heavy metals in plants [9] and fish [14], respectively. There is evidence that the composition and functioning of soil microbial communities change under eCO_2_ [15,16], which could be one potential mechanism how eCO_2_ will affect the environmental process of PAH accumulation in soil. Results of high throughput sequencing showed that eCO_2_ changed the phylogenetic diversity and richness of soil microbes (Fig 2), and shifted the composition of soil microbial communities (Fig 3). Moreover, eCO_2_ significantly decreased the frequency of functional genes which contribute to PAH degradation (Fig 4), providing a likely explanation for the enhancement of PAH accumulation in soil under eCO_2_. Although there are several abiotic degradation processes involved during the removal process of PAHs from soil, biodegradation is the major degradation process of PAHs [4]. This was confirmed by decreased mineralization of ^14^C-phenanthrene by fresh soil at eCO_2_ as compared to aCO_2_ in the present study (Fig 5).

In contrast to the significant effects on PAHs in soil, there was no significant difference in PAH concentration in rice seeds (*Oryza sativa* L. cv. Wu xiang jing 14) between aCO_2_ and eCO_2_ treatments (S2 Table). Nevertheless, given the significant effects of eCO_2_ on PAH concentrations in the soil, future studies should continue to investigate the effect of eCO_2_ on PAH accumulation in plants, especially in crops like rice and wheat that are key to human nutrition. Although there was no significant difference for this kind of rice, this outcome cannot be directly transferred to other crops. First, different crops differ in PAH accumulation [17]. Second, different plant species respond differently in their physiology to eCO_2_ [18], such as changes in stomatal conductance, which is likely to affect PAH accumulation in plant tissue. In addition, air-to-vegetation is the principal pathway for the accumulation of PAHs in plant shoots rather than soil-to-vegetation [17]. The FACE system we used already ran for more than 15 years, while rice grew only for ~130 days per year, which could explain why we found higher accumulation of PAHs in soil under eCO_2_, while no significant difference was found in seeds. Given the important implications of PAH accumulation in soil and crops for food safety and human health, further research is needed to explore plant-soil interactions of different crops.

A total of 3,500 different operational taxonomic units (OTUs) were identified in our bacterial community analysis. There was no significant difference in the phylogenetic diversity and richness of bacterial communities between eCO_2_ and aCO_2_ conditions (Fig 2), but the bacterial community structure of soil samples at eCO_2_ differed from that at aCO_2_ (Fig 3). Proteobacteria, Chloroflexi, Actinobacteria and Acidobacteria dominated the bacterial communities of both aCO_2_ and eCO_2_ treatments, adding up to 71.5 and 67.0% of the total bacterial OTUs, respectively (Fig 3). Elevated CO_2_ changed bacterial communities by significantly decreasing the relative abundance of Actinobacteria (P = 0.048).

A total of 2,395 different protistan OTUs were identified, and the effect of eCO_2_ on protistan communities was more pronounced compared to that on bacterial communities (Figs 2 and 3). In contrast to bacteria, eCO_2_ showed a clear tendency to decrease the phylogenetic diversity and richness of protistan communities, although this effect was not statistically significant (Fig 2). Similar to bacteria, eCO_2_ significantly altered the composition of protistan communities (Fig 3). Chloroplastida and fungi dominated the protistan communities of both aCO_2_ and eCO_2_ treatments, representing 66.1 and 68.5% of the total protistan OTUs, respectively (Fig 3). Elevated CO_2_ significantly increased the relative abundance of Chloroplastida (P = 0.022), while it decreased the relative abundance of fungi (P = 0.056).

Previous studies have shown responses of soil microbes to eCO_2_ to range from positive to negative [15,19,20]. These variable results indicate that eCO_2_ effects on soil microbial communities may depend on the environmental context, such as soil conditions and/or vegetation status [21]. In this study, eCO_2_ had no significant effect on the phylogenetic diversity and richness of bacteria, but tended to decrease the phylogenetic diversity and richness of protistans, and changed the community structure of both bacteria and protistans in the soil. We propose that the effect of elevated atmospheric CO_2_ on soil microbes may be due to changes in plant carbon inputs to the soil [22]. As the pool of labile soil carbon may be changed by an alteration of root exudation [23], this may lead to altered soil microbial activity [15, 20].

During the first 50 h, the mineralization of ^14^C-phenanthrene by fresh soils from the eCO_2_ treatment was similar to that of the aCO_2_ treatment, and the mineralization rate was slow (Fig 5). This may be due to the fact that soil microbes have to adapt to newly introduced organic pollutants first. After 100 h, mineralization rates of ^14^C-phenanthrene by fresh soils from eCO_2_ and aCO_2_ treatments were accelerated, and that in soil from the aCO_2_ treatment accelerated more rapidly (Fig 5). After 400 h, mineralization rates decelerated in soils from both aCO_2_ and eCO_2_ treatments, and relative differences between the treatments did not increase further (Fig 5).

The proportion of sequences contributing to PAH degradation decreased significantly at eCO_2_ compared to aCO_2_ (Fig 4) according to the predictive functional analysis [24]. In fact, the negative effect of eCO_2_ on PAH degradation may be due to the reduction of Actinobacteria and fungi at eCO_2_ (Fig 3), as both microbial groups are known to significantly contribute to PAH degradation [25].

## Conclusions

Our findings suggest that eCO_2_ changed the composition of soil microbial communities. Especially the eCO_2_-induced decrease of microbial groups being involved in PAH degradation may have resulted in PAH accumulation in soil at eCO_2_. Both a lower proportion of sequences contributing to PAH degradation and lower mineralization rates of ^14^C-phenanthrene at eCO_2_ indicate that eCO_2_ can accelerate PAH accumulation in soils. Although no significant difference in PAH concentration in rice seeds was observed, potential implications of eCO_2_ effects on PAH accumulation should be studied for food safety and human health in future environmental scenarios.

## Supporting information

S1 File Meta-data. (XLS)

S1 Table Comparison of PAH contents in soils under ambient and elevated CO_2_ conditions in 2015 and 2016. aCO_2_, ambient CO_2_; eCO_2_, elevated CO_2._ NA, AP, AC, F, Phe, Ant, Fl, Pyr, BaA, Chr, BbF, BkF, BaP, IP, DBahA, BghiP represent Naphthalene, Acenaphthylene, Acenaphthene, Fluorene, Phenanthrene, Anthracene, Fluoranthene, Pyrene, Benzo(a)anthracene, Chrysene, Benzo(b)fluoranthene, Benzo(k)fluoranthene, Benzo(a)pyrene, Indene(1,2,3-c,d)pyrene, Dibenzo(a,h)anthracene and Benzo(g,h,i)perylene, respectively.
(PDF)

S2 Table PAH contents and ANOVA analysis of seeds of rice grown under ambient and elevated CO_2_ conditions. aCO_2_, ambient CO_2_; eCO_2_, elevated CO_2._ NA, AP, AC, F, Phe, Ant, Fl, Pyr, BaA, Chr, BbF, BkF, BaP, IP, DBahA, BghiP represent Naphthalene, Acenaphthylene, Acenaphthene, Fluorene, Phenanthrene, Anthracene, Fluoranthene, Pyrene, Benzo(a)anthracene, Chrysene, Benzo(b)fluoranthene, Benzo(k)fluoranthene, Benzo(a)pyrene, Indene(1,2,3-c,d)pyrene, Dibenzo(a,h)anthracene and Benzo(g,h,i)perylene, respectively.
(PDF)
