# Supplementary material for: Elevated CO2 accelerates polycyclic aromatic hydrocarbon accumulation in a paddy soil grown with rice
Source: PLoS One. 2018 Apr 24;13(4):e0196439. doi: 10.1371/journal.pone.0196439 (PMC5916858; doi:10.1371/journal.pone.0196439)
Supplement: S2 Table — aCO2, ambient CO2; eCO2, elevated CO2. NA, AP, AC, F, Phe, Ant, Fl, Pyr, BaA, Chr, BbF, BkF, BaP, IP, DBahA, BghiP represent Naphthalene, Acenaphthylene, Acenaphthene, Fluorene, Phenanthrene, Anthracene, Fluoranthene, Pyrene, Benzo(a)anthracene, Chrysene, Benzo(b)fluoranthene, Benzo(k)fluoranthene, Benzo(a)pyrene, Indene(1,2,3-c,d)pyrene, Dibenzo(a,h)anthracene and Benzo(g,h,i)perylene, respectively. (PDF) [file pone.0196439.s003.pdf]

		NA	Ap	Ac	F	Phe	Ant	Fl	Pyr	BaA	Chr	BbF	BkF	BaP	Ip	DBahA	BghiP
**aCO_2**	**Mean**	7.62	1.40	0.68	5.58	32.50	30.95	0.83	2.89	4.88	4.18	0.32	N.D.	0.80	11.44	N.D.	0.55
	**SE**	0.38	0.21	0.08	0.61	4.38	4.16	0.57	0.06	0.26	0.79	0.05		0.00	1.06		0.14
**eCO_2**	**Mean**	7.29	1.10	0.54	4.72	31.24	29.77	1.73	3.02	5.23	4.61	0.38		0.83	7.57		0.87
	**SE**	0.38	0.05	0.06	0.63	3.49	3.32	0.48	0.38	1.07	1.12	0.07		0.27	1.88		0.28
	**F-value**	0.35	1.84	1.68	0.96	0.05	0.05	1.48	0.11	0.10	0.08	0.27		0.02	3.22		1.30
	**P-value**	0.58	0.25	0.29	0.38	0.83	0.84	0.31	0.76	0.77	0.80	0.64		0.91	0.22		0.34
